# Construction of a high-density linkage map and fine mapping of QTLs for growth and gonad related traits in blunt snout bream

**DOI:** 10.1038/srep46509

**Published:** 2017-04-19

**Authors:** Shi-Ming Wan, Hong Liu, Bo-Wen Zhao, Chun-Hong Nie, Wei-Min Wang, Ze-Xia Gao

**Affiliations:** 1 College of Fisheries, Key Lab of Agricultural Animal Genetics, Breeding and Reproduction of Ministry of Education/Key Lab of Freshwater Animal Breeding, Ministry of Agriculture, Huazhong Agricultural University, Wuhan, Hubei 430070, China; 2 Freshwater Aquaculture Collaborative Innovation Center of Hubei Province, Wuhan 430070, China; 3 Hubei Provincial Engineering Laboratory for Pond Aquaculture, Wuhan 430070, China

## Abstract

High-density genetic maps based on SNPs are essential for fine mapping loci controlling specific traits for fish species. Using restriction-site associated DNA tag sequencing (RAD-Seq) technology, we identified 42,784 SNPs evenly distributed across the *Megalobrama amblycephala* genome. Based on 2 parents and 187 intra-specific hybridization progenies, a total of 14,648 high-confidence SNPs were assigned to 24 consensus linkage groups (LGs) of maternal and paternal map. The total length of the integrated map was 3,258.38 cM with an average distance of 0.57 cM among 5676 effective loci, thereby representing the first high-density genetic map reported for *M. amblycephala*. A total of eight positive quantitative trait loci (QTLs) were detected in QTL analysis. Of that, five QTL explained ≥35% of phenotypic variation for growth traits and three QTL explained ≥16% phenotypic variation for gonad related traits. A total of 176 mapped markers had significant hits in the zebrafish genome and almost all of the 24 putative-chromosomes of *M. amblycephala* were in relatively conserved synteny with chromosomes of zebrafish. Almost all *M. amblycephala* and zebrafish chromosomes had a 1:1 correspondence except for putative-chromosome 4, which mapped to two chromosomes of zebrafish caused by the difference in chromosome numbers between two species.

Blunt snout bream (*Megalobrama amblycephala*, Yih, 1955), which distributes naturally in the middle and lower reaches of the Yangtze River in Central China, has been recognized as one of main aquaculture species in the freshwater pond polyculture system since 1960s^1^. The high nutritional value, ease of artificial propagation and high economic value has made *M. amblycephala* as one of the most important species in stock enhancement programs of China. In recent years, however, as a result of long term high-intensity stocking and lack of resource management, low growth rate and disease susceptibility has been reported recently^2^,^3^. Meanwhile, early puberty was also found in cultured *M. amblycephala* population at one year of age, which is normally reached by two or three years old in natural populations^4^. Early puberty adversely affects growth, feed utilization, health and welfare, which is a major problem in farmed fish^5^,^6^,^7^,^8^,^9^,^10^. Therefore, breeding new varieties is of great importance for *M. amblycephala* aquaculture. In order to enhance profitability and sustainability while maintaining genetic variability in the cultured stock, research related to growth and early puberty of *M. amblycephala* have been performed. Genetic parameters estimation for growth- and gonad-related traits as well as their correlations in *M. amblycephala* had been reported by our previously studies^11^,^12^. The genes and microRNAs involved in growth of *M. amblycephala* were also identified by high throughput transcriptome sequencing technology^2^,^3^. In addition, a batch of genes related to growth and gonad traits had been cloned and analyzed in *M. amblycephala*^13^,^14^,^15^. This information is useful for the germplasm evaluation and genetic analysis but is insufficient for fine mapping of loci or genes related to the important economic trait. Most of the traits targeted for species genetic enhancement are governed by multiple major and/or minor genes/QTLs (quantitative trait loci)^16^. Identification and fine mapping of genes underlying QTLs controlling important economic traits have been established as an effective approach for quantitative traits dissection in fish^17^. The development of genetic breeding programs based on marker/gene-assisted selection would benefit for *M. amblycephala* industry.

One of the baselines for efficient genetic selection programs is the availability of genetic linkage maps, which allow for mapping phenotypic traits of interest and provide a backbone for further genetic studies. Compared to traditional methods for linkage map construction, the newly developed genotyping by next-generation sequencing technologies, which allow the discovery and simultaneous scoring of thousands of single nucleotide polymorphism (SNP) markers from a single sequencing run for dozens of individuals, provide a new means to rapidly characterize the genomes of non-model species. SNP markers, representing the most abundant source of variation in the genome, are increasingly utilized for construction of high-density genetic linkage map^17^,^18^. Restriction-site associated DNA tag sequencing (RAD-Seq), as a reliable, high-throughput, affordable method to reduce genomic complexity, has been particularly attractive for SNP discovery and genotyping^19^. RAD-seq technology has been successfully applied in numerous fish species, such as Japanese flounder^17^, common pandora^20^, Asian seabass^21^, Atlantic salmon^22^, orange-spotted grouper^23^ and Sole^24^, for linkage map construction, evolutionary and comparative genomic analysis. The linkage map was then used for detection of QTLs related to economic traits in many fish species^25^,^26^,^27^,^28^. Six QTLs for growth traits and one candidate genes within significant QTL region were detected in Asian seabass^29^. Sex- and growth-related QTLs were detected based on a high-saturated RAD-Seq linkage map in turbot^30^.

Despite the commercial importance, so far limited effort has been invested in exploring the genomic structure of blunt snout bream. There is no available genetic linkage map for fine QTL mapping of interest traits and identification of candidate genes. In the present study, we consequently performed a large-scale identification of genome-wide SNPs derived from RAD-seq of a mapping population containing 2 parents and 187 intra-specific hybridization progenies. Then the first high-density SNP-based genetic linkage map of blunt snout bream was constructed using the results. We also successfully detected QTLs for the growth and gonad related traits in this bream. Finally, a chromosomal-level comparative analysis was performed between our SNP-based genetic map and zebra fish chromosome complement. Summarily, this map is useful in facilitating future assembly of the blunt snout bream genome, identification of QTLs for traits of interest and comparative genomics studies.

## Results

### RAD-seq library construction and sequencing

A total of 189 RAD-seq libraries from two parents and their 187 offspring were constructed and sequenced on an Illumina HiSeq2500 platform to generate raw reads. After data trimming, 922.99 million reads, comprising approximately 99.69 Gb of sequencing data, were individually partitioned into RAD tags according to their molecular-identifying sequence. Finally, female and male parental data sets, containing respectively 15.93 million filtered reads (comprising 1719.90 Mb of data with a GC% of 37.06) and 14.71 million filtered reads (comprising 1588.68 Mb of data with a GC% of 37.05), were correspondingly partitioned into 13.68 and 12,62 million RAD tags. These RAD tags were aligned and clustered into 327,364 and 323,929 stacks, respectively, and 31,149 and 31,509 candidate alleles were inferred (Supplementary Table S1). From the 187 offspring, a total of 892.35 million filtered reads (average of 4.77 million) corresponding to 96,378.27 Mb of data (average of 515.39 Mb) were produced and divided into 697,396,575 RAD tags (ranging from 1,140,488 to 6,621,573 with an average of 3,729,393) to construct stacks for individual SNP discovery (Supplementary Table S1). The data also showed the parents were sequenced at a substantially higher depth than the offspring to maximize the probability of SNPs detection in the parents. Our sequences are available at the NCBI Short Read Archive (http://www.ncbi.nlm.nih.gov/Traces/sra/), at accession SRS1797758.

### SNP discovery and genotyping

**Figure 1:**
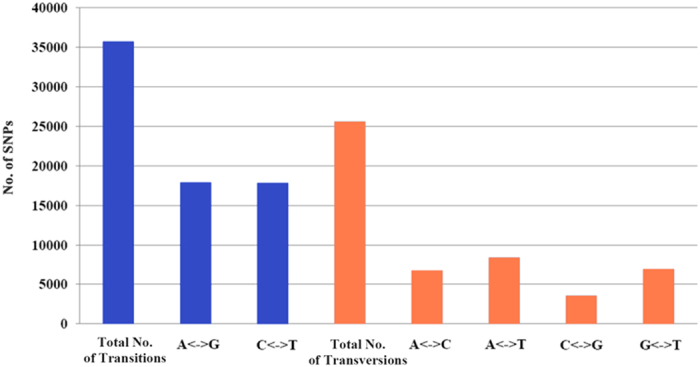
Transitions and transversions within 61,284 biallelic SNPs detected among parents.

After stringent selection, the parental RAD tags were assembled into 367,640 contigs with an average length of 374 bp (Table 1), which showed a high assembly quality used for SNP assays and subsequent genotyping. A total of 61,284 putative SNPs between two parents were then detected using the criteria described before (sequencing depth ≥5 and ≤200, base quality ≥25). The nucleotide variation of these SNPs in *M. amblycephala* parents were biallelic and consisted of 58% transitions and 42% transversions with a ratio of 1.40 (Fig. 1, Supplementary Table S2). Subsequently, stacks were used for genotyping across 187 offspring following the criteria described above. A total of 14,648 high-fidelity SNPs with fixed genotypes in both parents were retained after excluding those with a deviation for the Mendelian segregation pattern, and alleles of each marker were assigned to their respective parental donor. The flanking sequences of all 14,648 SNPs were listed in Supplementary Table S3.

### High-density genetic map construction

**Figure 2:**
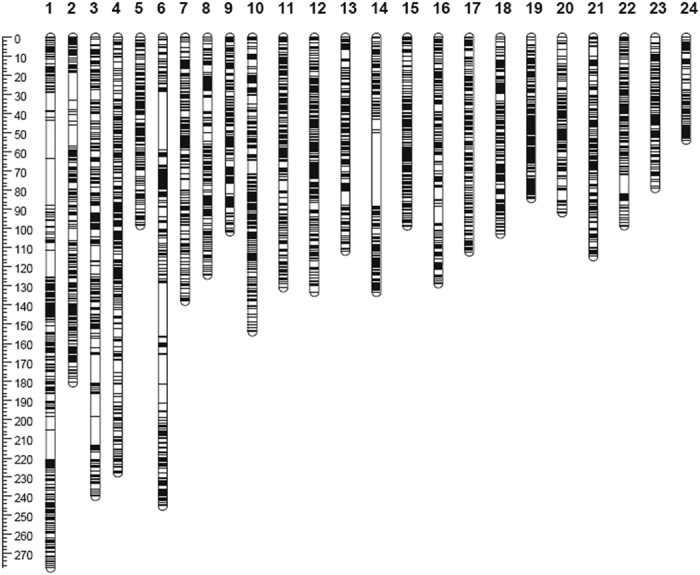
Linkage group lengths and marker distribution of the high-resolution restriction site-associated DNA sequencing-based SNP genetic map of *M. amblycephala*.

Based on a double pseudo-test cross strategy, a high-density RAD-based SNP genetic map of *M. amblycephala* was first constructed using Lep-MAP. A total of 14,648 segregating SNPs were successfully classified into 24 LGs (Table 2). The maternal map contained 9,531 SNPs with a total genetic distance of 2390.06 cM; the length of each LG ranged from 45.28 cM (LG24) to 177.37 cM (LG11), with an average genetic length of 99.59 cM (Table 2). The corresponding paternal map consisted of 9,847 SNPs representing a total length of 2109.82 cM and ranging from 44.97 cM (LG8) to 196.19 cM (LG1) with an average genetic length of 87.91 cM (Table 2). Interestingly, LG24 contained the least number of SNP and showed the shortest length for both of the parents. 170 SNPs with a distance of 45.28 cM and 147 SNPs with a distance of 44.97 cM were observed in maternal and paternal map, respectively. LG1 was the biggest LG in paternal map (196.19 cM) and the second biggest LG in maternal map (176.24 cM). But LG11 of paternal map only had a distance of 79.66 cM, which was significantly shorter. Finally, we constructed an integrated map consisting of 24 LGs, which corresponded to 5,676 effective loci including 14,648 SNPs (Table 2, Fig. 2 and Supplementary Fig. S1). The total length of the integrated map was 3258.38 cM with an average number of effective loci of 237 spanning 145.72 cM and an average inter-locus distance of 0.56 cM. The genetic length of each LG ranged from 53.47 cM (LG24) to 277.38 cM (LG1), with an average inter-locus distance of 0.39–0.88 cM. The densest LG was LG4, which contained 382 effective loci with an average density of 0.6 cM, whereas LG24 had the least amount of effective loci (only 121) (Table 2). Names and positions of markers on the 24 LGs of the integrated genetic map were listed in Supplementary Table S4.

### Growth and gonad associated QTLs

**Figure 3:**
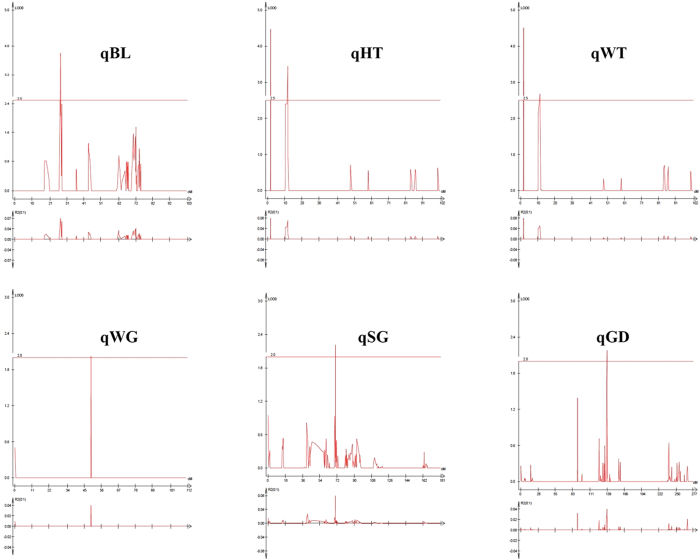
Growth and gonad related trait associated QTLs found in *M. amblycephala*.

Eight significant QTLs for body length (BL), height (HT), weight (WT), weight of gonad (WG), stage of gonad development (SG) and gender (GD) were distributed on LG1, LG2, LG9, LG13 and LG18 of *M. amblycephala*, respectively (Table 3 and Fig. 3). Among them, five QTLs for BL, HT and WT were detected with LOD ≥ 2.5 and three QTL for WG, SG and GD were detected with LOD ≥ 2. Half of these QTLs were identified on LG9. One cluster containing two QTLs (qHT-2 and qWT-2) was detected between the narrow positions of 11.2–12.4 cM on LG9. These two adjacent QTLs were associated with different traits. The qHT-1 and qWT-1 located to the same position of LG9 contained same associated marker. These two QTLs located at 1.8–2.0 cM of LG9 had the highest LOD value of 4.48, and correspondingly had the highest contribution to phenotypic variation of 8%. On LG18, QTL for BL (qBL) with a LOD value of 3.8 and explained 7% of the phenotypic variation. Three QTLs (qWG, qSG and qGD) related to gonad development were detected around 49.2, 70.2 and 138.0 cM on LG13, LG2 and LG1, respectively, with a contribution to phenotypic variation of 4%, 8% and 4% (Table 3 and Fig. 3). Five QTLs explained ≥35% of phenotypic variation for growth, and 3 QTLs explained ≥16% phenotypic variation for gonad were detected. No major loci explaining >10% of the total variation were detected. The fact that these loci do not independently have higher contributions to such a complicated trait is not unexpected. The polymorphic SNPs marking QTLs and their association sequence are shown in Supplementary Table S3.

### Comparative genome analysis

**Figure 4:**
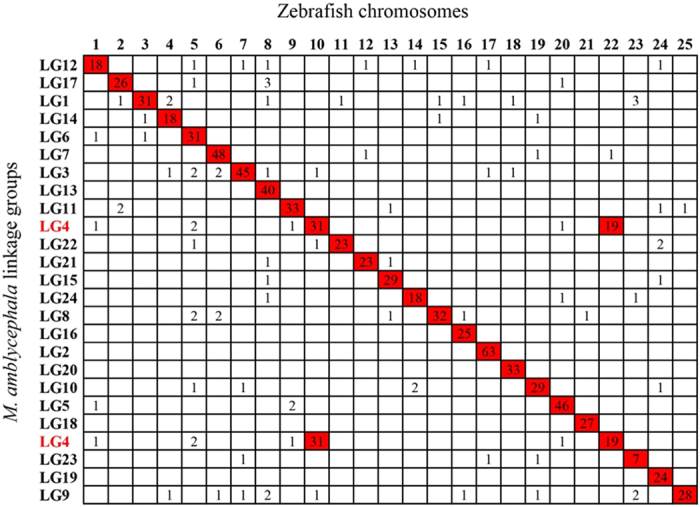
Homologous chromosome relationships between *M. amblycephala* and *D. rerio*. The numbers in the cells are the orthologous genes between *M. amblycephala* and *D. rerio* located on the designated chromosome (LG).

In total, 24 putative-chromosomes were constructed based on the LGs; each chromosome comprised a mean linkage distance of 135.77 cM (Table 3). Following this, we compared the chromosomal orders of marker sequences of blunt snout bream with their counterparts in zebrafish. A total of 176 mapped markers had significant hits in the whole genome sequences of zebrafish. As shown in Fig. 4, all of the 24 mamc (mamc: *M. amblycephala* chromosome) were in relatively conserved synteny with drec (drec: *Danio rerio* chromosome). Due to the inconsistency of chromosome number, mamc4 was found to have 31 and 19 syntenic blocks with drec10 and drec22, respectively. Of all, mamc2 exhibited the highest degrees of synteny with 63 hits on drec17. Similarly, mamc7, mamc3, mamc13 and mamc5 were also found to be highly syntenic with drec6, drec7, drec8 and drec12. The results of this comparison suggest that *M. amblycephala* is very closely related to zebrafish.

## Discussion

High-density genetic linkage maps with molecular markers are important for genomic and genetic analyses of individual species. In present study, we constructed the first high-resolution genetic map of *M. amblycephala* using SNPs, which represent the most common type of genome DNA polymorphism and are amenable to high-throughput genotyping^31^. Finally, an integrated map consisting of 24 LGs, which corresponded to 5,676 effective loci, was constructed based on 14,648 SNPs. A total length of the integrated map was 3258.38 cM with an average inter-locus distance of 0.56 cM. The genetic length of each LG ranged from 53.47 cM to 277.38 cM, with an average inter-locus distance of 0.39–0.88 cM. These data provides a good resource for future genome selection and genome-wide association studies. Compared with previous fish genetic maps, our study also showed a significant progress in map quality. In Atlantic salmon, 6,458 SNPs were initially assigned to 32 linkage groups with an average of 220 SNPs per linkage group and the total map length for each mapping parent was from 1426 cM to 2807 cM^21^. The sex-averaged map of orange-spotted grouper contained a total of 4,608 SNPs, which spanned 1581.7 cM, with an average inter-location space at 0.56 cM and a mean distance between SNPs of 0.34 cM^22^. In Asian seabass, a high density genetic map was constructed by 3321 SNPs with a total length of 1577.67 cM and an average marker interval of 0.52 cM^29^. A genetic linkage map consisting of 867 SNP markers on 22 linkage groups spanning 1130.63 cM with the mean marker spacing of 1.30 cM was obtained in cichlid fishes^32^. Kakioka *et al*. constructed a gudgeons linkage map spanning 1,390.9 cM with 25 linkage groups and an average marker interval of 0.87 cM based on 1,622 RAD-tag markers^33^. The first genetic linkage map for *M. amblycephala* presented an obvious progress on the number of markers, length and average marker interval of linkage map comparing these fish species. Only the genetic map of Japanese flounder showed a similar quality, with 12,712 high-confidence SNPs of total length of 3,497.29 cM and average distance of 0.47 cM between loci^17^.

Using Lep-MAP, SNPs were successfully classified into 24 LGs for *M. amblycephala*. LGs for the paternal map had a similar length to those of the maternal map. For instance, LG24 contained of the least number of SNPs and showed the shortest maternal and paternal maps. LG1 was the biggest LG in the paternal map and the second biggest LG in the maternal map. But, the length of LG11 showed a noteworthy inconsistency between maternal (177.37 cM) and paternal maps (79.66 cM). Similar phenomenon has been found in other species, with for example more than one pair of corresponding LGs showing different length maternal and paternal maps for Japanese flounder^17^. In order to explore whether it is related to gender, population size or sequencing error, additional markers need to be developed and more research is required.

The high-density linkage map generated in our study provided a platform for QTL fine mapping for economically important traits of *M. amblycephala*. Growth^3^ is one of the most important economic parameters for cultured fish species. QTLs for growth traits have been detected in some fish species based on corresponding linkage maps. In turbot, a total of 220 QTLs associated with two body length traits and two body weight traits in different growth periods were detected, which explained the corresponding phenotypic variance, ranging from 14.4–100%^30^. In European and Asian seabass, 38 and 6 QTLs for growth traits and corresponding candidate genes within the significant QTL region were detected respectively^24^,^29^. Moreover, QTLs for body-weight and other growth-related traits were also detected in Atlantic salmon^26^, bighead carp^27^, goldfish^28^ and other important economic fish species. In the present study, five significant QTLs associated with growth-related traits with LOD ≥ 2.5 were detected in *M. amblycephala*. Of them, two QTLs (qHT-2 and qWT-2) associated with height and weight were identified on LG9, mapping to one cluster. The quite short genetic and physical distances between the QTLs mapping to this cluster suggest that the individual cluster is closely related to growth, which also appeared in Japanese flounder that six QTLs were concentrated within a narrow region on LG6 and three were found in another cluster on LG21^17^. Moreover, the marker RAD257309 located in LG9 was associated to qHT-1 and qWT-1 simultaneously (Table 3). These two QTLs had the highest LOD value of 4.48, and correspondingly had the highest contribution to phenotypic variation of 8%. These indicated that gene sequences around the marker may be closely related to growth traits. A single locus may affect two or more distinct phenotypic, which was defined as pleiotropy^34^ and had been reported in fish species. For instance, in European grayling, temperature-driven gene expression changes in fish adapted to differing thermal environments are constrained by the level of gene pleiotropy^35^. Weight and height were important phenotypes with positive correlation used in condition assessment of fish^36^. In our study, these two traits (HT and WT) were traced to a SNP polymorphism, which provides a support for the pleiotropy and a good example for analysis of genotype and phenotypic. Although no major loci explaining >10% of the total variation were detected, 5 QTLs explained ≥35% of phenotypic variation for growth-related traits of *M. amblycephala*. Two patterns of growth-related QTL in fish have been reported in previous studies. One is single major QTL accounted for significant portion of a trait and the other is many QTLs with small or intermediate effects on the trait. Most of studies uncovered that multiple QTLs influenced growth in fish, such as in bighead carp^27^ and stickleback^37^. The result from our present study is consistent with these fish species.

As early puberty can adversely affect growth, Gutierrez *et al*. identified one QTL related to late sexual maturation in Atlantic salmon^38^. In our study, two QTLs related to weight (qWG) and development stage (qSG) of gonad at one-year-old *M. amblycephala* were detected on LG13 and LG2, respectively. The qWG and qSG could be useful for *M. amblycephala* breeding to avoid individuals with overdevelopment gonad and early maturation in muscle-increasing stage. QTL analysis also provided basic genetic information for sex determination. In previous studies, Lee *et al*. identified important markers linked to sex-determining loci in tilapia (*Oreochromis* spp.)^39^. Palaiokostas *et al*. also detected a novel sex-determining locus in Nile tilapia through QTL analysis^40^. It is reasonable to believe that the qGD detected for *M. amblycephala* in the present study could be useful to identify its sex determination genes in the future.

This study is the first attempt to identify the QTLs for *M. amblycephala*. Further experiments need to be implemented to verify our findings and enhance the scale and quality of detected QTLs. The polymorphic SNPs included in QTLs and their associated sequence from our research will be useful for genome alignment, gene function analysis and genetic improvement cooperating with conventional breeding technologies.

Through comparing genome sequence, we can understand the differences and similarities of nucleotide composition, linear relationship and gene order between different species sequenced, thereby providing useful information related to genetic analysis prediction, gene location and biological system evolutionary relationships and so on. The different genomes of two species evolved from a common ancestor genome; the closer the two organisms on the evolutionary stage, the higher their genome correlation. If evolutionary relationship was extremely close, genomes between two organisms will show synteny, which can be used for gene mapping, gene function annotation, phylogenetic analysis and genome structure analysis^41^,^42^,^43^. Next-generation sequencing technology has enabled the generation of draft genomes for individual species as well as rapid development of comparative analyses among multiple genomes based on chromosomal-assembly levels^17^,^44^. Linkage map with sequence-based markers is a platform for comparative genome analysis. In the present study, 24 putative-chromosomes of *M. amblycephala* were constructed using a high-density genetic map. We compared the chromosomal orders of marker sequences of *M. amblycephala* with their counterparts in *D. rerio*. A total of 176 mapped markers had significant hits in the zebrafish genome and all of the 24 mamc were in relatively conserved synteny with drec. Almost all *M. amblycephala* and *D. rerio* chromosomes had a 1:1 correspondence except for that mamc4 was found to have 31 and 19 syntenic blocks with drec10 and drec22, respectively, which caused by the difference in chromosome numbers among two fish species (*D. rerio* has 25 chromosomes^45^, whereas *M. amblycephala* has 24). The results of this comparison not only supports the marker ordering on the map but also facilitates the functional inference of genes in *M. amblycephala* and further benefits the researches of comparative genomics and genome evolution.

## Materials and Methods

All animals and experiments were conducted in accordance with the “Guidelines for Experimental Animals” of the Ministry of Science and Technology (Beijing, China). The study was approved by the Institutional Animal Care and Use Ethics Committee of Huazhong Agricultural University. All efforts were made to minimize suffering. All experimental procedures involving fish were approved by the institution animal care and use committee of the Huazhong Agricultural University.

### Mapping Population and DNA Extraction

The F1 mapping population consists of 187 progenies from an inter-specific cross between two heterozygous genotypes. Individual favors rapid/slow growth was used as the female/male parent. Two parents and the progenies were bred in fish breeding base of Huazhong Agricultural University (Huanggang, China) in 2014. One-year-old growth traits including body length (BL), height (HT), weight (WT) and gonad traits including weight of gonad (WG), stage of gonad development (SG)^4^ and gender (GD, ♀/♂) were measured and recorded for all progenies. Genomic DNA was isolated from the fins of the parental fish and 187 progenies using traditional phenol-chloroform extraction in combination with RNase treatment. All DNA samples were quantified using a NanoDrop instrument (Thermo Scientific, Wilmington, DE, USA) and quality assessed by 1% agarose gel electrophoresis.

### Library Construction and RAD Tag Sequencing

Individual genomic DNA (0.3–1.0 μg) was digested for 15 min at 37 °C in a 50 mL reaction with 20 units (U) of EcoR I (New England Biolabs). The methods of RAD library construction had been reported by Baird *et al*.^46^ except 4–8 bp long specific nucleotide barcodes were ligated to Illumina P1 adapter and were used to distinguish different samples. Adapter-ligated fragments were then pooled and randomly sheared to DNA fragments with average size of 500 bp (Bioruptor Branson sonicator 450). After end repair, 3′-adenine overhangs were added, a paired-end P2 adapter containing T overhangs was ligated. Finally, libraries were subjected to PCR enrichment and RAD for each individual were sequenced on an Illumina Hiseq 2500 platform using paired-end reads (125 bp) (BGI, Shenzhen, China).

### RAD Sequence Data Analysis

After removing adapters and low quality bases (the reads containing more than 50% quality value ≤5) from the sequenced data, the 4–8 bp long specific nucleotide barcodes and specific recognition site (AATTC) was used to assigned the reads to different individuals with one base mismatch. Reads that did not match unique barcodes and specific sequence were discarded. Nine hundred and twenty-three million clean reads were then further trimmed to RAD tags with uniform length of 117 nucleotides (nt), which comprised the 5 nt of *Eco*R I recognition site and the 112 nt of potentially variable sequence.

The parental RAD paired-end data was assembled by the software of Rainbow 2.02^47^. Contigs with length below 200 bp were also removed. In order to obtain the SNPs of parents, the parents’ paired-end RAD reads were mapped onto the reference contigs by SOAP2.20^48^. SNPs were detected by SOAPsnp^49^ to calculate the likelihood of each genotype in each individual. A threshold of 200 for sequencing depth was used to remove overexpression RAD sequences, which are likely to be genome repetitive sequences^50^. Potential high-quality SNP markers, which genotypes were different between two parents, were identified following the criteria (sequencing depth ≥5 and ≤200, base quality ≥25). But the total number of parents SNPs showed a substantial increase at the tails of the sequences (the last nine nucleotides from the positions 109 to 117) (Supplementary Fig. S2), which was sequencing errors as described previously^51^ and consequently removed from the analyses. And the first paired-end reads are more suitable for detecting SNPs because of a higher coverage than the second reads and have more fixed sequences^50^. Thus only the first (left) paired-read with a length of 108 nt was used in subsequent analyses. Stacks (version 0.9998)^52^ was then used for RAD sequences assembly, alleles and SNP discovery for the clean reads. Algorithm as follows: 1. RAD tags reads that match exactly in parent or progenies were clustered into stacks with a minimum stack depth of 5. A maximum sequence mismatch of 2 was allowed to merge into a locus between stacks within an individual; 2. A maximum sequence difference of 3 was allowed to create a catalogue of the parents’ possible loci and alleles; 3. Each individual was matched against the catalogue. Finally, a minimum stack depth of 10 reads was used to create a stack in an individual for genotyping, automated corrections options were set as minimum stack depth of 10 to be called as homozygous.

### Linkage Map Construction and QTL

A double pseudo-test cross strategy was used for genetic map construction^53^. The linkage analysis was done using JoinMap 4.0 software with CP population type codes^54^. The separation type of markers was divided into three types: hk × hk (Parents were heterozygous), lm × ll (Female parent was heterozygous, male parent was homozygous recessiveness) and nn × np (Female parent was homozygous recessiveness, male parent was heterozygous). The hk × hk Markers were expected to segregate in a 1:2:1 ratio and lm × ll or nn × np markers were expected to segregate in a 1:1 ratio. The ratio of marker segregation was calculated by Chi-square test. Markers with significantly distorted segregation (P-value < 0.01) will be excluded from the map construction. The separate chromosomes module with a logarithm of odds (LOD) score limit of 12 was then used to obtain the linkage group assignments. The marker order of paternal and maternal maps was obtained using the order markers module. An integrated sex averaged linkage map was also obtained using this software.

The composite interval mapping (CIM) algorithm implemented in the WinQTLCart2.5 software was used for QTL analysis^55^. The CIM analysis was based on the Model 6 with four parameters (a 10-cM window size, five control markers, a 1-cM step size and forward and backward stepwise regression). LOD scores were calculated every cM with 1,000 permutations tests at a whole genome-wide significance level of P < 0.05. a conservative LOD threshold of 2.5 and 2.0 were used to declare significance of QTL.

### Comparative genome analysis

Consensus sequences of the mapped RAD tag (108 bases in length) were aligned with the genomic sequences of zebrafish (Zv9), which has public genome sequence information. BLAST (BLAST^+^, version 2.2.21)^56^ alignment between two species was performed with an e-value cutoff of 10^−10^. The smallest e-value was used to define a significant hit to eliminate the interference of a query sequence hit two or more loci. Significant hits including unoriented scaffolds assigned to the chromosomes of zebrafish were used in the construction of Oxford grids^57^, which was then used to study genome synteny and to compare positions of the homologous loci using Grid Map ver. 3.0a (http://cbr.jic.ac.uk/dicks/software/Grid_Map).

## Conclusion

RAD-seq technology was used for large-scale identification of SNPs that were then successfully used for high-throughput genotyping and construction of a high-density genetic linkage map of *M. amblycephala*. The generated genetic map is the first genetic map to date for *M. amblycephala*. Through SNP mapping analysis, we identified eight positive QTLs associated with growth- and gonad-related traits for *M. amblycephala*. We also anchored the genome sequences to putative-chromosomes and further identified a conservative syntenic relationship between *M. amblycephala* and *D. rerio* by comparative genomic analysis. The large numbers of genome polymorphism SNPs and the high-density genetic map not only lays the foundation for chromosomal-level analysis of the *M. amblycephala* genome but also provides an excellent genetic resource for future marker-assisted selection breeding.

## Additional Information

**How to cite this article:** Wan, S.-M. *et al*. Construction of a high-density linkage map and fine mapping of QTLs for growth and gonad related traits in blunt snout bream. *Sci. Rep.* **7**, 46509; doi: 10.1038/srep46509 (2017).

**Publisher's note:** Springer Nature remains neutral with regard to jurisdictional claims in published maps and institutional affiliations.

## Supplementary Material

Supplementary Figures

Supplementary Tables

## Figures and Tables

**Table 1:** RAD paired-end contig assembly of the parents.

	Contig length (bp)	Number
N50	404	164,632
N60	399	198,905
N70	390	233,770
N80	364	269,928
N90	288	312,940
Total length	137,629,749	
Maximum length	1,826	
Number >= 200 bp		367,640
Average length	374	
GC rate (%)	0.372	

**Table 2:** The summary of the genetic linkage map constructed in *M. amblycephala*.

Linkage Group	Maternal map		Paternal map		Integrated map			
	No. of SNPs	Distance (cM)	No. of SNPs	Distance (cM)	No. of SNPs	No. of effective loci	Distance (cM)	Average inter-loci distance (cM)
1	575	176.24	584	196.19	964	350	277.38	0.79
2	708	138.92	286	85.62	879	310	180.51	0.58
3	548	154.32	570	124.56	858	317	239.92	0.76
4	430	156.88	561	143.28	822	382	227.7	0.60
5	356	83.01	547	76.23	730	215	98.28	0.46
6	414	112.2	472	93.96	678	277	244.96	0.88
7	455	99.32	358	101.46	661	251	137.85	0.55
8	329	80.61	505	80.09	658	234	124.21	0.53
9	407	73.29	475	61	645	208	101.57	0.49
10	372	133.28	430	100.67	643	259	153.87	0.59
11	423	177.37	400	79.66	641	265	130.86	0.49
12	413	125.78	424	91.95	610	281	133.32	0.47
13	426	71.9	476	71.57	605	215	111.71	0.52
14	472	68.37	350	75.69	603	226	133.3	0.59
15	359	73.66	382	85.59	577	201	98.45	0.49
16	377	77.43	391	77.86	572	239	128.81	0.54
17	471	84.2	430	91.76	557	190	112.09	0.59
18	384	82.22	333	77.65	547	220	102.77	0.47
19	248	83.42	423	62.32	516	218	84.11	0.39
20	362	72	390	76.34	459	168	91.56	0.55
21	263	75.92	292	77.01	417	210	114.46	0.55
22	282	70.25	307	68.21	417	180	98.37	0.55
23	287	74.19	314	66.18	343	139	78.85	0.57
24	170	45.28	147	44.97	246	121	53.47	0.44
**Total**	**9531**	**2390.06**	**9847**	**2,109.82**	**14648**	**5676**	**3258.38**	**Mean 0.57**

**Table 3:** Characteristics of growth and gonad associated QTLs.

QTL	Linkage group	Genetic position	Associated marker	LOD	R^2^
qBL	LG18	27.1–27.4	RAD15371	3.8	0.07
qHT-1	LG9	1.8–2.0	RAD257309	4.48	0.08
qHT-2	LG9	11.7–12.4	RAD236205	3.41	0.07
qWT-1	LG9	1.8–2.0	RAD257309	4.48	0.08
qWT-2	LG9	11.2–11.3	RAD82931	2.65	0.05
qWG	LG13	49.2–49.3	RAD49930	2.01	0.04
qSG	LG2	70.3–70.4	RAD5610	2.2	0.08
qGD	LG1	137.6–139.1	RAD212933	2.18	0.04

R^2^, explained phenotypic variation.
