# Supplementary material for: Construction of a high-density linkage map and fine mapping of QTLs for growth and gonad related traits in blunt snout bream
Source: Sci Rep. 2017 Apr 19;7:46509. doi: 10.1038/srep46509 (PMC5395971; doi:10.1038/srep46509)
Supplement: Supplementary Figures [file srep46509-s1.pdf]

Construction of a high-density linkage map and fine mapping of QTL for growth and gonad related traits in blunt snout bream

Shi-Ming Wan[1,2], Hong Liu[1,2], Bo-Wen Zhao[1,2], Chun-Hong Nie[1,2], Wei-Min Wang[1], Ze-Xia Gao[1,2,3*]

[1] College of Fisheries, Key Lab of Agricultural Animal Genetics, Breeding and Reproduction of Ministry of Education/Key Lab of Freshwater Animal Breeding, Ministry of Agriculture, Huazhong Agricultural University, Wuhan, Hubei 430070, China

[2] Freshwater Aquaculture Collaborative Innovation Center of Hubei Province, Wuhan 430070, China

[3] Hubei Provincial Engineering Laboratory for Pond Aquaculture, Wuhan 430070, China

*Corresponding author: Ze-Xia Gao, College of Fisheries, Huazhong Agricultural University, Wuhan, 430070 Hubei, China. E-mail address: gaozexia@hotmail.com

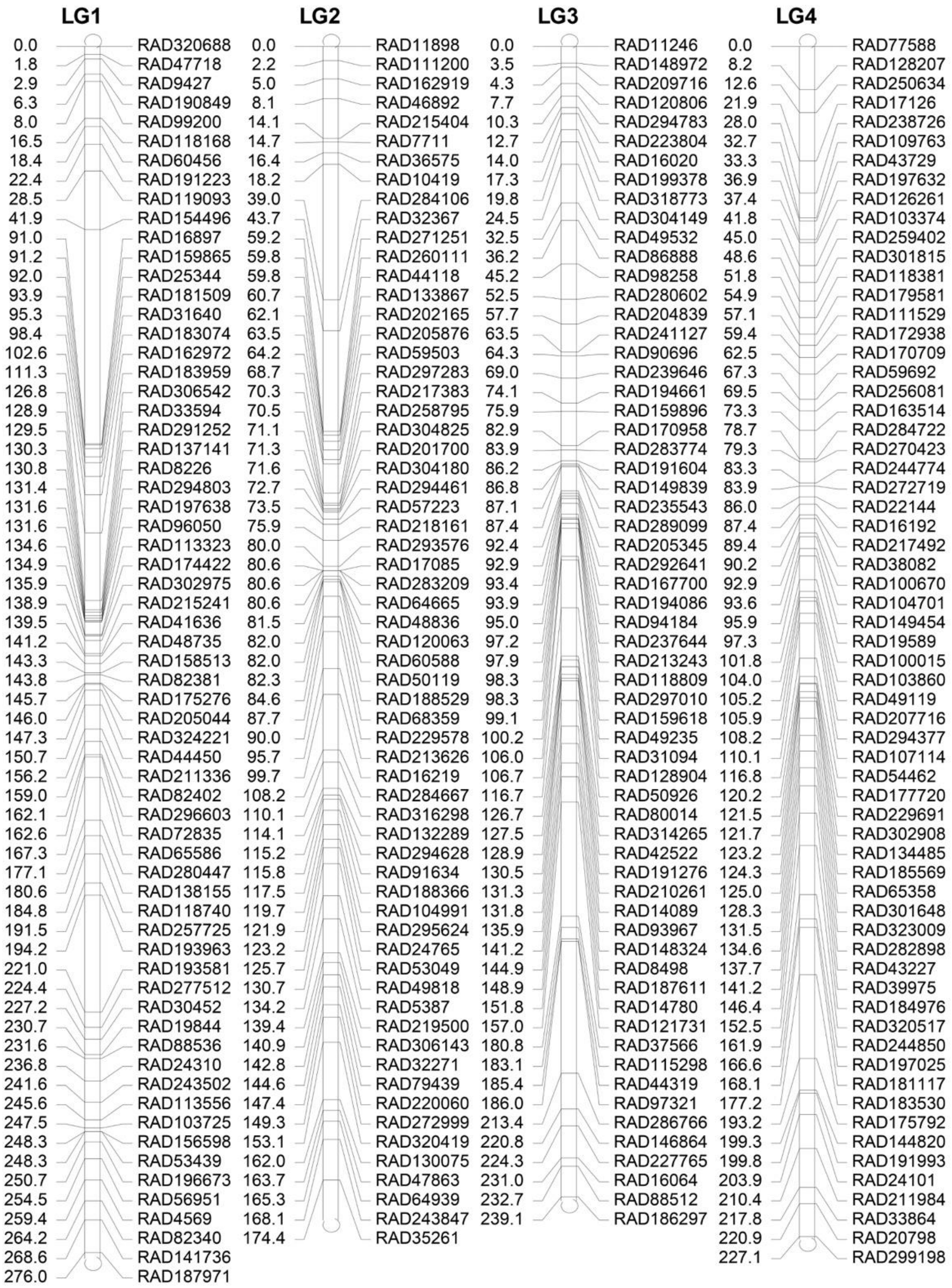
LG1
0.0 RAD320688
1.8 RAD47718
2.9 RAD9427
6.3 RAD190849
8.0 RAD99200
16.5 RAD118168
18.4 RAD60456
22.4 RAD191223
28.5 RAD119093
41.9 RAD154496
91.0 RAD16897
91.2 RAD159865
92.0 RAD25344
93.9 RAD181509
95.3 RAD31640
98.4 RAD183074
102.6 RAD162972
111.3 RAD183959
126.8 RAD306542
128.9 RAD33594
129.5 RAD291252
130.3 RAD137141
130.8 RAD8226
131.4 RAD294803
131.6 RAD197638
131.6 RAD96050
134.6 RAD113323
134.9 RAD174422
135.9 RAD302975
138.9 RAD215241
139.5 RAD41636
141.2 RAD48735
143.3 RAD158513
143.8 RAD82381
145.7 RAD175276
146.0 RAD205044
147.3 RAD324221
150.7 RAD44450
156.2 RAD211336
159.0 RAD82402
162.1 RAD296603
162.6 RAD72835
167.3 RAD65586
177.1 RAD280447
180.6 RAD138155
184.8 RAD118740
191.5 RAD257725
194.2 RAD193963
221.0 RAD193581
224.4 RAD277512
227.2 RAD30452
230.7 RAD19844
231.6 RAD88536
236.8 RAD24310
241.6 RAD243502
245.6 RAD113556
247.5 RAD103725
248.3 RAD156598
248.3 RAD53439
250.7 RAD196673
254.5 RAD56951
259.4 RAD4569
264.2 RAD82340
268.6 RAD141736
276.0 RAD187971
LG2
0.0 RAD11898
2.2 RAD111200
5.0 RAD162919
8.1 RAD46892
14.1 RAD215404
14.7 RAD7711
16.4 RAD36575
18.2 RAD10419
39.0 RAD284106
43.7 RAD32367
59.2 RAD271251
59.8 RAD260111
59.8 RAD44118
60.7 RAD133867
62.1 RAD202165
63.5 RAD205876
64.2 RAD59503
68.7 RAD297283
70.3 RAD217383
70.5 RAD258795
71.1 RAD304825
71.3 RAD201700
71.6 RAD304180
72.7 RAD294461
73.5 RAD57223
75.9 RAD218161
80.0 RAD293576
80.6 RAD17085
80.6 RAD283209
80.6 RAD64665
81.5 RAD48836
82.0 RAD120063
82.0 RAD60588
82.3 RAD50119
84.6 RAD188529
87.7 RAD68359
90.0 RAD229578
95.7 RAD213626
99.7 RAD16219
108.2 RAD284667
110.1 RAD316298
114.1 RAD132289
115.2 RAD294628
115.8 RAD91634
117.5 RAD188366
119.7 RAD104991
121.9 RAD295624
123.2 RAD24765
125.7 RAD53049
130.7 RAD49818
134.2 RAD5387
139.4 RAD219500
140.9 RAD306143
142.8 RAD32271
144.6 RAD79439
147.4 RAD220060
149.3 RAD272999
153.1 RAD320419
162.0 RAD130075
163.7 RAD47863
165.3 RAD64939
168.1 RAD243847
174.4 RAD35261
LG3
0.0 RAD11246
3.5 RAD148972
4.3 RAD209716
7.7 RAD120806
10.3 RAD294783
12.7 RAD223804
14.0 RAD16020
17.3 RAD199378
19.8 RAD318773
24.5 RAD304149
32.5 RAD49532
36.2 RAD86888
45.2 RAD98258
52.5 RAD280602
57.7 RAD204839
63.5 RAD241127
64.3 RAD90696
69.0 RAD239646
74.1 RAD194661
75.9 RAD159896
82.9 RAD170958
83.9 RAD283774
86.2 RAD191604
86.8 RAD149839
87.1 RAD235543
87.4 RAD289099
92.4 RAD205345
92.9 RAD292641
93.4 RAD167700
93.9 RAD194086
95.0 RAD94184
97.2 RAD237644
97.9 RAD213243
98.3 RAD118809
98.3 RAD297010
99.1 RAD159618
100.2 RAD49235
106.0 RAD31094
106.7 RAD128904
116.7 RAD50926
126.7 RAD80014
127.5 RAD314265
128.9 RAD42522
130.5 RAD191276
131.3 RAD210261
131.8 RAD14089
135.9 RAD93967
141.2 RAD148324
144.9 RAD8498
148.9 RAD187611
151.8 RAD14780
157.0 RAD121731
180.8 RAD37566
183.1 RAD115298
185.4 RAD44319
186.0 RAD97321
213.4 RAD286766
220.8 RAD146864
224.3 RAD227765
231.0 RAD16064
232.7 RAD88512
239.1 RAD186297
LG4
0.0 RAD77588
8.2 RAD128207
12.6 RAD250634
21.9 RAD17126
28.0 RAD238726
32.7 RAD109763
33.3 RAD43729
36.9 RAD197632
37.4 RAD126261
41.8 RAD103374
45.0 RAD259402
48.6 RAD301815
51.8 RAD118381
54.9 RAD179581
57.1 RAD111529
59.4 RAD172938
62.5 RAD170709
67.3 RAD59692
69.5 RAD256081
73.3 RAD163514
78.7 RAD284722
79.3 RAD270423
83.3 RAD244774
83.9 RAD272719
86.0 RAD22144
87.4 RAD16192
89.4 RAD217492
90.2 RAD38082
92.9 RAD100670
93.6 RAD104701
95.9 RAD149454
97.3 RAD19589
101.8 RAD100015
104.0 RAD103860
105.2 RAD49119
105.9 RAD207716
108.2 RAD294377
110.1 RAD107114
116.8 RAD54462
120.2 RAD177720
121.5 RAD229691
121.7 RAD302908
123.2 RAD134485
124.3 RAD185569
125.0 RAD65358
128.3 RAD301648
131.5 RAD323009
134.6 RAD282898
137.7 RAD43227
141.2 RAD39975
146.4 RAD184976
152.5 RAD320517
161.9 RAD244850
166.6 RAD197025
168.1 RAD181117
177.2 RAD183530
193.2 RAD175792
199.3 RAD144820
199.8 RAD191993
203.9 RAD24101
210.4 RAD211984
217.8 RAD33864
220.9 RAD20798
227.1 RAD299198

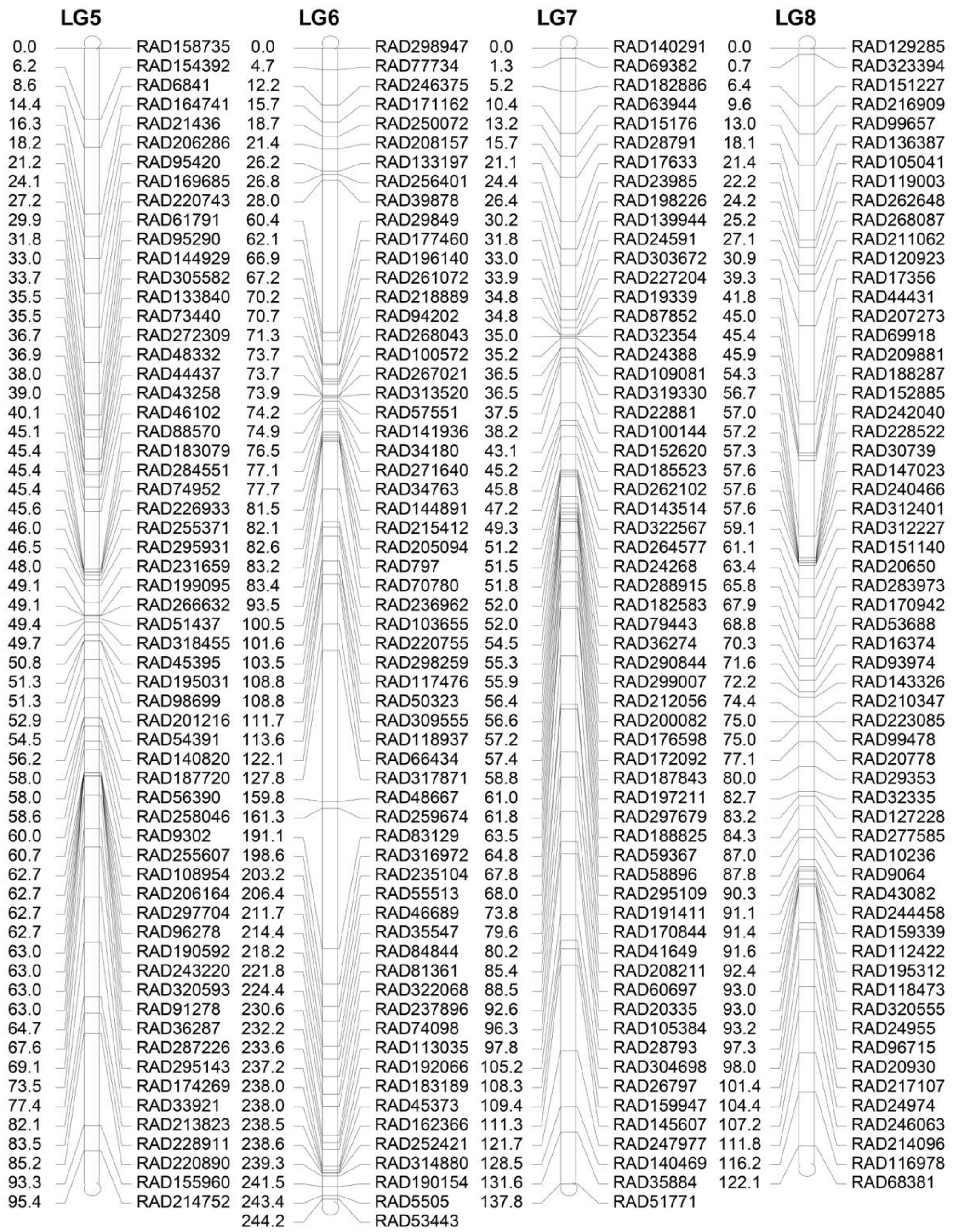
LG5
0.0 RAD158735
6.2 RAD154392
8.6 RAD6841
14.4 RAD164741
16.3 RAD21436
18.2 RAD206286
21.2 RAD95420
24.1 RAD169685
27.2 RAD220743
29.9 RAD61791
31.8 RAD95290
33.0 RAD144929
33.7 RAD305582
35.5 RAD133840
35.5 RAD73440
36.7 RAD272309
36.9 RAD48332
38.0 RAD44437
39.0 RAD43258
40.1 RAD46102
45.1 RAD88570
45.4 RAD183079
45.4 RAD284551
45.4 RAD74952
45.6 RAD226933
46.0 RAD255371
46.5 RAD295931
48.0 RAD231659
49.1 RAD199095
49.1 RAD266632
49.4 RAD51437
49.7 RAD318455
50.8 RAD45395
51.3 RAD195031
51.3 RAD98699
52.9 RAD201216
54.5 RAD54391
56.2 RAD140820
58.0 RAD187720
58.0 RAD56390
58.6 RAD258046
60.0 RAD9302
60.7 RAD255607
62.7 RAD108954
62.7 RAD206164
62.7 RAD297704
62.7 RAD96278
63.0 RAD190592
63.0 RAD243220
63.0 RAD320593
63.0 RAD91278
64.7 RAD36287
67.6 RAD287226
69.1 RAD295143
73.5 RAD174269
77.4 RAD33921
82.1 RAD213823
83.5 RAD228911
85.2 RAD220890
93.3 RAD155960
95.4 RAD214752
LG6
0.0 RAD298947
4.7 RAD77734
12.2 RAD246375
15.7 RAD171162
18.7 RAD250072
21.4 RAD208157
26.2 RAD133197
26.8 RAD256401
28.0 RAD39878
60.4 RAD29849
62.1 RAD177460
66.9 RAD196140
67.2 RAD261072
70.2 RAD218889
70.7 RAD94202
71.3 RAD268043
73.7 RAD100572
73.7 RAD267021
73.9 RAD313520
74.2 RAD57551
74.9 RAD141936
76.5 RAD34180
77.1 RAD271640
77.7 RAD34763
81.5 RAD144891
82.1 RAD215412
82.6 RAD205094
83.2 RAD797
83.4 RAD70780
93.5 RAD236962
100.5 RAD103655
101.6 RAD220755
103.5 RAD298259
108.8 RAD117476
108.8 RAD50323
111.7 RAD309555
113.6 RAD118937
122.1 RAD66434
127.8 RAD317871
159.8 RAD48667
161.3 RAD259674
191.1 RAD83129
198.6 RAD316972
203.2 RAD235104
206.4 RAD55513
211.7 RAD46689
214.4 RAD35547
218.2 RAD84844
221.8 RAD81361
224.4 RAD322068
230.6 RAD237896
232.2 RAD74098
233.6 RAD113035
237.2 RAD192066
238.0 RAD183189
238.0 RAD45373
238.5 RAD162366
238.6 RAD252421
239.3 RAD314880
241.5 RAD190154
243.4 RAD5505
244.2 RAD53443
LG7
0.0 RAD140291
1.3 RAD69382
5.2 RAD182886
10.4 RAD63944
13.2 RAD15176
15.7 RAD28791
21.1 RAD17633
24.4 RAD23985
26.4 RAD198226
30.2 RAD139944
31.8 RAD24591
33.0 RAD303672
33.9 RAD227204
34.8 RAD19339
34.8 RAD87852
35.0 RAD32354
35.2 RAD24388
36.5 RAD109081
36.5 RAD319330
37.5 RAD22881
38.2 RAD100144
43.1 RAD152620
45.2 RAD185523
45.8 RAD262102
47.2 RAD143514
49.3 RAD322567
51.2 RAD264577
51.5 RAD24268
51.8 RAD288915
52.0 RAD182583
52.0 RAD79443
54.5 RAD36274
55.3 RAD290844
55.9 RAD299007
56.4 RAD212056
56.6 RAD200082
57.2 RAD176598
57.4 RAD172092
58.8 RAD187843
61.0 RAD197211
61.8 RAD297679
63.5 RAD188825
64.8 RAD59367
67.8 RAD58896
68.0 RAD295109
73.8 RAD191411
79.6 RAD170844
80.2 RAD41649
85.4 RAD208211
88.5 RAD60697
92.6 RAD20335
96.3 RAD105384
97.8 RAD28793
105.2 RAD304698
108.3 RAD26797
109.4 RAD159947
111.3 RAD145607
121.7 RAD247977
128.5 RAD140469
131.6 RAD35884
137.8 RAD51771
LG8
0.0 RAD129285
0.7 RAD323394
6.4 RAD151227
9.6 RAD216909
13.0 RAD99657
18.1 RAD136387
21.4 RAD105041
22.2 RAD119003
24.2 RAD262648
25.2 RAD268087
27.1 RAD211062
30.9 RAD120923
39.3 RAD17356
41.8 RAD44431
45.0 RAD207273
45.4 RAD69918
45.9 RAD209881
54.3 RAD188287
56.7 RAD152885
57.0 RAD242040
57.2 RAD228522
57.3 RAD30739
57.6 RAD147023
57.6 RAD240466
57.6 RAD312401
59.1 RAD312227
61.1 RAD151140
63.4 RAD20650
65.8 RAD283973
67.9 RAD170942
68.8 RAD53688
70.3 RAD16374
71.6 RAD93974
72.2 RAD143326
74.4 RAD210347
75.0 RAD223085
75.0 RAD99478
77.1 RAD20778
80.0 RAD29353
82.7 RAD32335
83.2 RAD127228
84.3 RAD277585
87.0 RAD10236
87.8 RAD9064
90.3 RAD43082
91.1 RAD244458
91.4 RAD159339
91.6 RAD112422
92.4 RAD195312
93.0 RAD118473
93.0 RAD320555
93.2 RAD24955
97.3 RAD96715
98.0 RAD20930
101.4 RAD217107
104.4 RAD24974
107.2 RAD246063
111.8 RAD214096
116.2 RAD116978
122.1 RAD68381

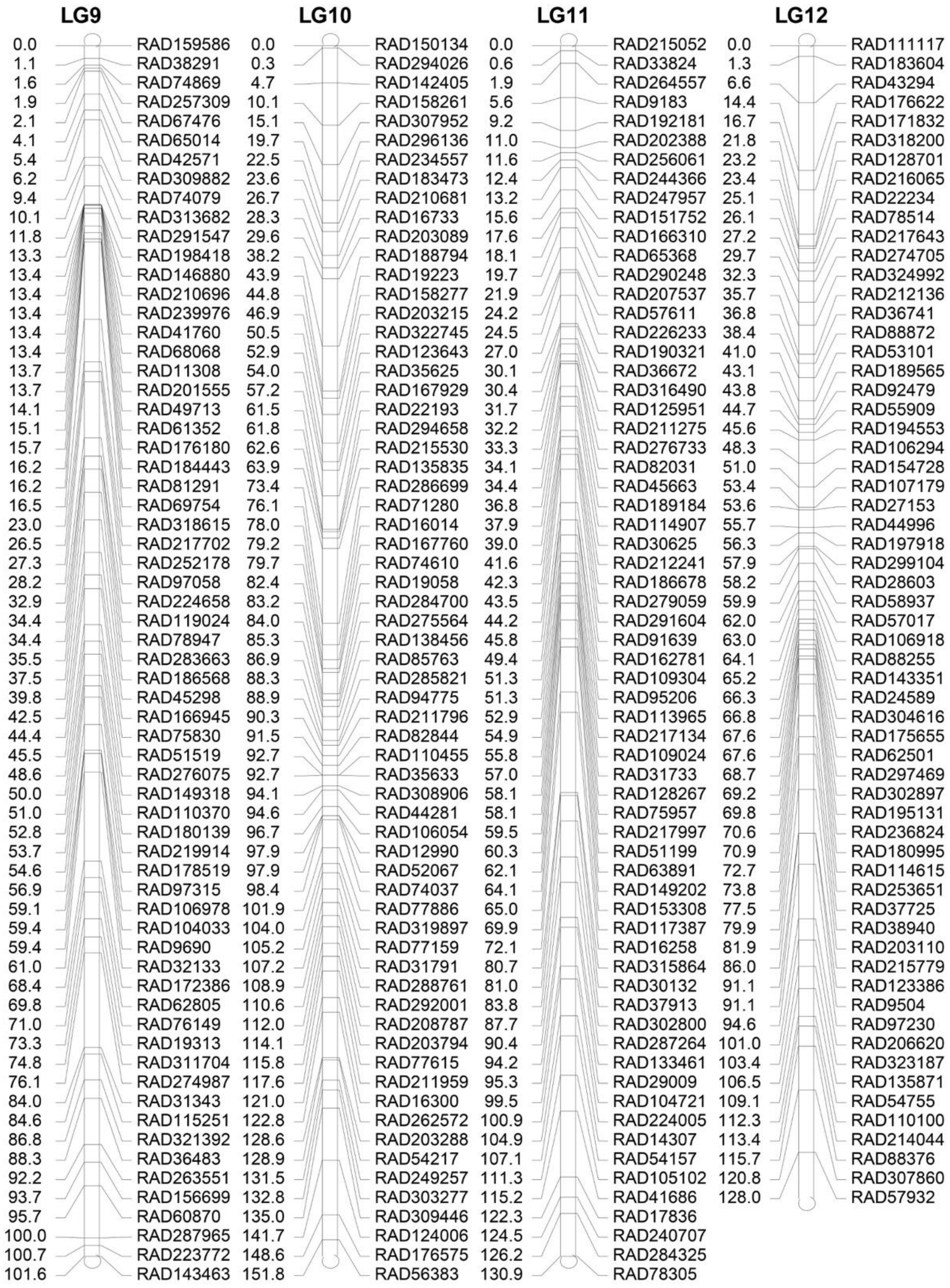
LG9
0.0 RAD159586
1.1 RAD38291
1.6 RAD74869
1.9 RAD257309
2.1 RAD67476
4.1 RAD65014
5.4 RAD42571
6.2 RAD309882
9.4 RAD74079
10.1 RAD313682
11.8 RAD291547
13.3 RAD198418
13.4 RAD146880
13.4 RAD210696
13.4 RAD239976
13.4 RAD41760
13.4 RAD68068
13.7 RAD11308
13.7 RAD201555
14.1 RAD49713
15.1 RAD61352
15.7 RAD176180
16.2 RAD184443
16.2 RAD81291
16.5 RAD69754
23.0 RAD318615
26.5 RAD217702
27.3 RAD252178
28.2 RAD97058
32.9 RAD224658
34.4 RAD119024
34.4 RAD78947
35.5 RAD283663
37.5 RAD186568
39.8 RAD45298
42.5 RAD166945
44.4 RAD75830
45.5 RAD51519
48.6 RAD276075
50.0 RAD149318
51.0 RAD110370
52.8 RAD180139
53.7 RAD219914
54.6 RAD178519
56.9 RAD97315
59.1 RAD106978
59.4 RAD104033
59.4 RAD9690
61.0 RAD32133
68.4 RAD172386
69.8 RAD62805
71.0 RAD76149
73.3 RAD19313
74.8 RAD311704
76.1 RAD274987
84.0 RAD31343
84.6 RAD115251
86.8 RAD321392
88.3 RAD36483
92.2 RAD263551
93.7 RAD156699
95.7 RAD60870
100.0 RAD287965
100.7 RAD223772
101.6 RAD143463
LG10
0.0 RAD150134
0.3 RAD294026
4.7 RAD142405
10.1 RAD158261
15.1 RAD307952
19.7 RAD296136
22.5 RAD234557
23.6 RAD183473
26.7 RAD210681
28.3 RAD16733
29.6 RAD203089
38.2 RAD188794
43.9 RAD19223
44.8 RAD158277
46.9 RAD203215
50.5 RAD322745
52.9 RAD123643
54.0 RAD35625
57.2 RAD167929
61.5 RAD22193
61.8 RAD294658
62.6 RAD215530
63.9 RAD135835
73.4 RAD286699
76.1 RAD71280
78.0 RAD16014
79.2 RAD167760
79.7 RAD74610
82.4 RAD19058
83.2 RAD284700
84.0 RAD275564
85.3 RAD138456
86.9 RAD85763
88.3 RAD285821
88.9 RAD94775
90.3 RAD211796
91.5 RAD82844
92.7 RAD110455
92.7 RAD35633
94.1 RAD308906
94.6 RAD44281
96.7 RAD106054
97.9 RAD12990
97.9 RAD52067
98.4 RAD74037
101.9 RAD77886
104.0 RAD319897
105.2 RAD77159
107.2 RAD31791
108.9 RAD288761
110.6 RAD292001
112.0 RAD208787
114.1 RAD203794
115.8 RAD77615
117.6 RAD211959
121.0 RAD16300
122.8 RAD262572
128.6 RAD203288
128.9 RAD54217
131.5 RAD249257
132.8 RAD303277
135.0 RAD309446
141.7 RAD124006
148.6 RAD176575
151.8 RAD56383
LG11
0.0 RAD215052
0.6 RAD33824
1.9 RAD264557
5.6 RAD9183
9.2 RAD192181
11.0 RAD202388
11.6 RAD256061
12.4 RAD244366
13.2 RAD247957
15.6 RAD151752
17.6 RAD166310
18.1 RAD65368
19.7 RAD290248
21.9 RAD207537
24.2 RAD57611
24.5 RAD226233
27.0 RAD190321
30.1 RAD36672
30.4 RAD316490
31.7 RAD125951
32.2 RAD211275
33.3 RAD276733
34.1 RAD82031
34.4 RAD45663
36.8 RAD189184
37.9 RAD114907
39.0 RAD30625
41.6 RAD212241
42.3 RAD186678
43.5 RAD279059
44.2 RAD291604
45.8 RAD91639
49.4 RAD162781
51.3 RAD109304
51.3 RAD95206
52.9 RAD113965
54.9 RAD217134
55.8 RAD109024
57.0 RAD31733
58.1 RAD128267
58.1 RAD75957
59.5 RAD217997
60.3 RAD51199
62.1 RAD63891
64.1 RAD149202
65.0 RAD153308
69.9 RAD117387
72.1 RAD16258
80.7 RAD315864
81.0 RAD30132
83.8 RAD37913
87.7 RAD302800
90.4 RAD287264
94.2 RAD133461
95.3 RAD29009
99.5 RAD104721
100.9 RAD224005
104.9 RAD14307
107.1 RAD54157
111.3 RAD105102
115.2 RAD41686
122.3 RAD17836
124.5 RAD240707
126.2 RAD284325
130.9 RAD78305
LG12
0.0 RAD111117
1.3 RAD183604
6.6 RAD43294
14.4 RAD176622
16.7 RAD171832
21.8 RAD318200
23.2 RAD128701
23.4 RAD216065
25.1 RAD22234
26.1 RAD78514
27.2 RAD217643
29.7 RAD274705
32.3 RAD324992
35.7 RAD212136
36.8 RAD36741
38.4 RAD88872
41.0 RAD53101
43.1 RAD189565
43.8 RAD92479
44.7 RAD55909
45.6 RAD194553
48.3 RAD106294
51.0 RAD154728
53.4 RAD107179
53.6 RAD27153
55.7 RAD44996
56.3 RAD197918
57.9 RAD299104
58.2 RAD28603
59.9 RAD58937
62.0 RAD57017
63.0 RAD106918
64.1 RAD88255
65.2 RAD143351
66.3 RAD24589
66.8 RAD304616
67.6 RAD175655
67.6 RAD62501
68.7 RAD297469
69.2 RAD302897
69.8 RAD195131
70.6 RAD236824
70.9 RAD180995
72.7 RAD114615
73.8 RAD253651
77.5 RAD37725
79.9 RAD38940
81.9 RAD203110
86.0 RAD215779
91.1 RAD123386
91.1 RAD9504
94.6 RAD97230
101.0 RAD206620
103.4 RAD323187
106.5 RAD135871
109.1 RAD54755
112.3 RAD110100
113.4 RAD214044
115.7 RAD88376
120.8 RAD307860
128.0 RAD57932

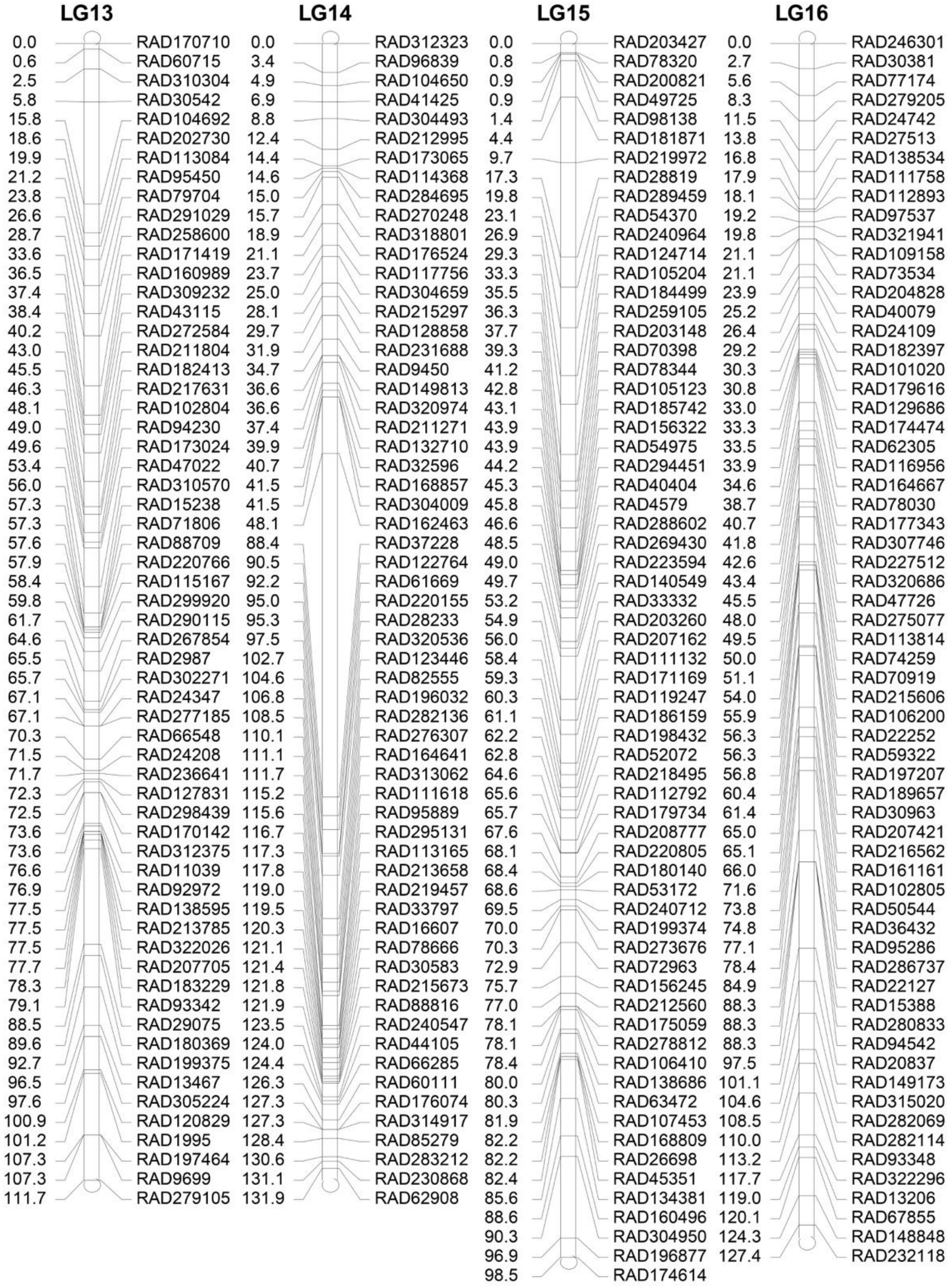

LG13
0.0 RAD170710
0.6 RAD60715
2.5 RAD310304
5.8 RAD30542
15.8 RAD104692
18.6 RAD202730
19.9 RAD113084
21.2 RAD95450
23.8 RAD79704
26.6 RAD291029
28.7 RAD258600
33.6 RAD171419
36.5 RAD160989
37.4 RAD309232
38.4 RAD43115
40.2 RAD272584
43.0 RAD211804
45.5 RAD182413
46.3 RAD217631
48.1 RAD102804
49.0 RAD94230
49.6 RAD173024
53.4 RAD47022
56.0 RAD310570
57.3 RAD15238
57.3 RAD71806
57.6 RAD88709
57.9 RAD220766
58.4 RAD115167
59.8 RAD299920
61.7 RAD290115
64.6 RAD267854
65.5 RAD2987
65.7 RAD302271
67.1 RAD24347
67.1 RAD277185
70.3 RAD66548
71.5 RAD24208
71.7 RAD236641
72.3 RAD127831
72.5 RAD298439
73.6 RAD170142
73.6 RAD312375
76.6 RAD11039
76.9 RAD92972
77.5 RAD138595
77.5 RAD213785
77.5 RAD322026
77.7 RAD207705
78.3 RAD183229
79.1 RAD93342
88.5 RAD29075
89.6 RAD180369
92.7 RAD199375
96.5 RAD13467
97.6 RAD305224
100.9 RAD120829
101.2 RAD1995
107.3 RAD197464
107.3 RAD9699
111.7 RAD279105
LG14
0.0 RAD312323
3.4 RAD96839
4.9 RAD104650
6.9 RAD41425
8.8 RAD304493
12.4 RAD212995
14.4 RAD173065
14.6 RAD114368
15.0 RAD284695
15.7 RAD270248
18.9 RAD318801
21.1 RAD176524
23.7 RAD117756
25.0 RAD304659
28.1 RAD215297
29.7 RAD128858
31.9 RAD231688
34.7 RAD9450
36.6 RAD149813
36.6 RAD320974
37.4 RAD211271
39.9 RAD132710
40.7 RAD32596
41.5 RAD168857
41.5 RAD304009
48.1 RAD162463
88.4 RAD37228
90.5 RAD122764
92.2 RAD61669
95.0 RAD220155
95.3 RAD28233
97.5 RAD320536
102.7 RAD123446
104.6 RAD82555
106.8 RAD196032
108.5 RAD282136
110.1 RAD276307
111.1 RAD164641
111.7 RAD313062
115.2 RAD111618
115.6 RAD95889
116.7 RAD295131
117.3 RAD113165
117.8 RAD213658
119.0 RAD219457
119.5 RAD33797
120.3 RAD16607
121.1 RAD78666
121.4 RAD30583
121.8 RAD215673
121.9 RAD88816
123.5 RAD240547
124.0 RAD44105
124.4 RAD66285
126.3 RAD60111
127.3 RAD176074
127.3 RAD314917
128.4 RAD85279
130.6 RAD283212
131.1 RAD230868
131.9 RAD62908
LG15
0.0 RAD203427
0.8 RAD78320
0.9 RAD200821
0.9 RAD49725
1.4 RAD98138
4.4 RAD181871
9.7 RAD219972
17.3 RAD28819
19.8 RAD289459
23.1 RAD54370
26.9 RAD240964
29.3 RAD124714
33.3 RAD105204
35.5 RAD184499
36.3 RAD259105
37.7 RAD203148
39.3 RAD70398
41.2 RAD78344
42.8 RAD105123
43.1 RAD185742
43.9 RAD156322
43.9 RAD54975
44.2 RAD294451
45.3 RAD40404
45.8 RAD4579
46.6 RAD288602
48.5 RAD269430
49.0 RAD223594
49.7 RAD140549
53.2 RAD33332
54.9 RAD203260
56.0 RAD207162
58.4 RAD111132
59.3 RAD171169
60.3 RAD119247
61.1 RAD186159
62.2 RAD198432
62.8 RAD52072
64.6 RAD218495
65.6 RAD112792
65.7 RAD179734
67.6 RAD208777
68.1 RAD220805
68.4 RAD180140
68.6 RAD53172
69.5 RAD240712
70.0 RAD199374
70.3 RAD273676
72.9 RAD72963
75.7 RAD156245
77.0 RAD212560
78.1 RAD175059
78.1 RAD278812
78.4 RAD106410
80.0 RAD138686
80.3 RAD63472
81.9 RAD107453
82.2 RAD168809
82.2 RAD26698
82.4 RAD45351
85.6 RAD134381
88.6 RAD160496
90.3 RAD304950
96.9 RAD196877
98.5 RAD174614
LG16
0.0 RAD246301
2.7 RAD30381
5.6 RAD77174
8.3 RAD279205
11.5 RAD24742
13.8 RAD27513
16.8 RAD138534
17.9 RAD111758
18.1 RAD112893
19.2 RAD97537
19.8 RAD321941
21.1 RAD109158
21.1 RAD73534
23.9 RAD204828
25.2 RAD40079
26.4 RAD24109
29.2 RAD182397
30.3 RAD101020
30.8 RAD179616
33.0 RAD129686
33.3 RAD174474
33.5 RAD62305
33.9 RAD116956
34.6 RAD164667
38.7 RAD78030
40.7 RAD177343
41.8 RAD307746
42.6 RAD227512
43.4 RAD320686
45.5 RAD47726
48.0 RAD275077
49.5 RAD113814
50.0 RAD74259
51.1 RAD70919
54.0 RAD215606
55.9 RAD106200
56.3 RAD22252
56.3 RAD59322
56.8 RAD197207
60.4 RAD189657
61.4 RAD30963
65.0 RAD207421
65.1 RAD216562
66.0 RAD161161
71.6 RAD102805
73.8 RAD50544
74.8 RAD36432
77.1 RAD95286
78.4 RAD286737
84.9 RAD22127
88.3 RAD15388
88.3 RAD280833
88.3 RAD94542
97.5 RAD20837
101.1 RAD149173
104.6 RAD315020
108.5 RAD282069
110.0 RAD282114
113.2 RAD93348
117.7 RAD322296
119.0 RAD13206
120.1 RAD67855
124.3 RAD148848
127.4 RAD232118

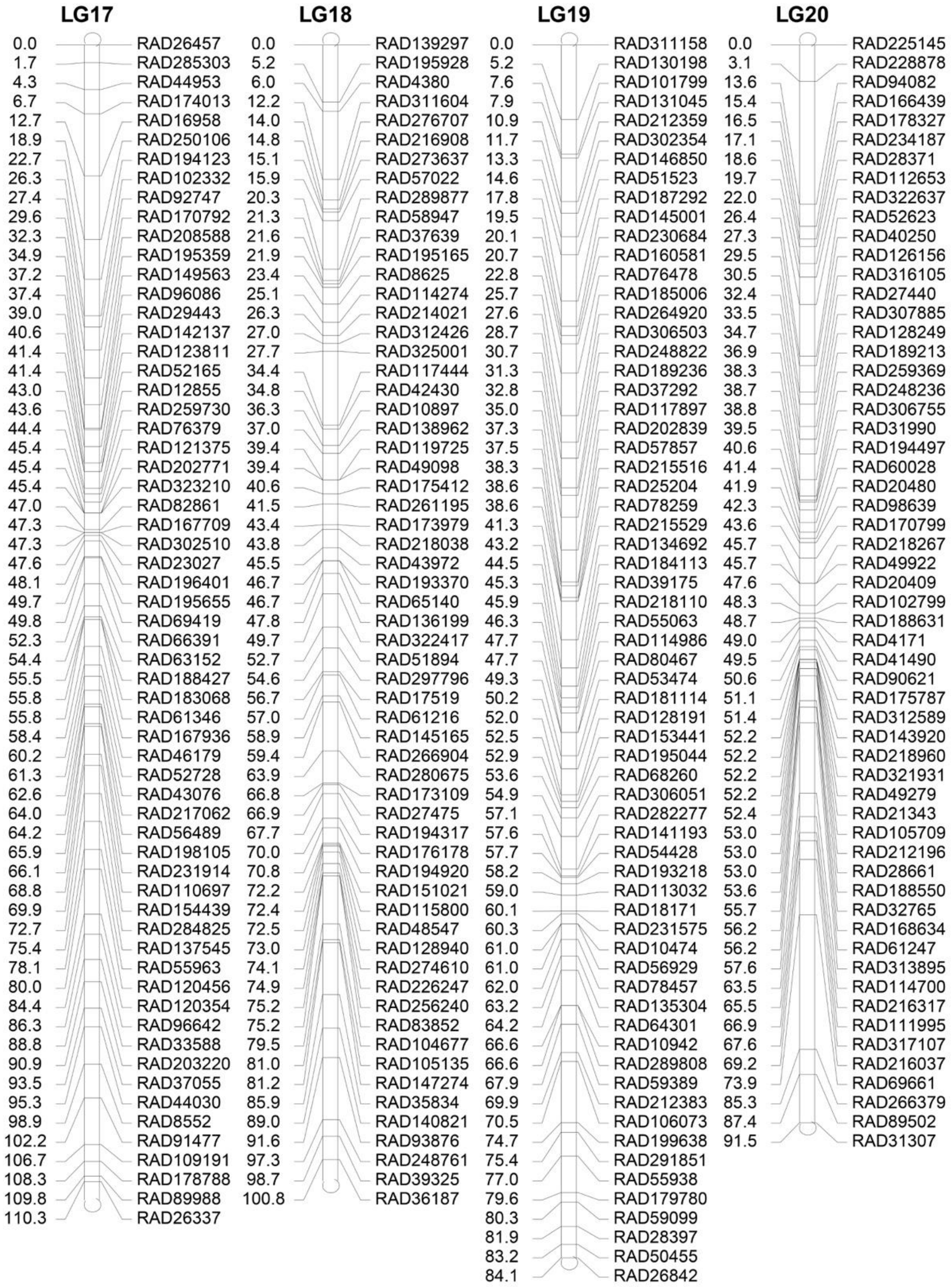
LG17
0.0 RAD26457
1.7 RAD285303
4.3 RAD44953
6.7 RAD174013
12.7 RAD16958
18.9 RAD250106
22.7 RAD194123
26.3 RAD102332
27.4 RAD92747
29.6 RAD170792
32.3 RAD208588
34.9 RAD195359
37.2 RAD149563
37.4 RAD96086
39.0 RAD29443
40.6 RAD142137
41.4 RAD123811
41.4 RAD52165
43.0 RAD12855
43.6 RAD259730
44.4 RAD76379
45.4 RAD121375
45.4 RAD202771
45.4 RAD323210
47.0 RAD82861
47.3 RAD167709
47.3 RAD302510
47.6 RAD23027
48.1 RAD196401
49.7 RAD195655
49.8 RAD69419
52.3 RAD66391
54.4 RAD63152
55.5 RAD188427
55.8 RAD183068
55.8 RAD61346
58.4 RAD167936
60.2 RAD46179
61.3 RAD52728
62.6 RAD43076
64.0 RAD217062
64.2 RAD56489
65.9 RAD198105
66.1 RAD231914
68.8 RAD110697
69.9 RAD154439
72.7 RAD284825
75.4 RAD137545
78.1 RAD55963
80.0 RAD120456
84.4 RAD120354
86.3 RAD96642
88.8 RAD33588
90.9 RAD203220
93.5 RAD37055
95.3 RAD44030
98.9 RAD8552
102.2 RAD91477
106.7 RAD109191
108.3 RAD178788
109.8 RAD89988
110.3 RAD26337
LG18
0.0 RAD139297
5.2 RAD195928
6.0 RAD4380
12.2 RAD311604
14.0 RAD276707
14.8 RAD216908
15.1 RAD273637
15.9 RAD57022
20.3 RAD289877
21.3 RAD58947
21.6 RAD37639
21.9 RAD195165
23.4 RAD8625
25.1 RAD114274
26.3 RAD214021
27.0 RAD312426
27.7 RAD325001
34.4 RAD117444
34.8 RAD42430
36.3 RAD10897
37.0 RAD138962
39.4 RAD119725
39.4 RAD49098
40.6 RAD175412
41.5 RAD261195
43.4 RAD173979
43.8 RAD218038
45.5 RAD43972
46.7 RAD193370
46.7 RAD65140
47.8 RAD136199
49.7 RAD322417
52.7 RAD51894
54.6 RAD297796
56.7 RAD17519
57.0 RAD61216
58.9 RAD145165
59.4 RAD266904
63.9 RAD280675
66.8 RAD173109
66.9 RAD27475
67.7 RAD194317
70.0 RAD176178
70.8 RAD194920
72.2 RAD151021
72.4 RAD115800
72.5 RAD48547
73.0 RAD128940
74.1 RAD274610
74.9 RAD226247
75.2 RAD256240
75.2 RAD83852
79.5 RAD104677
81.0 RAD105135
81.2 RAD147274
85.9 RAD35834
89.0 RAD140821
91.6 RAD93876
97.3 RAD248761
98.7 RAD39325
100.8 RAD36187
LG19
0.0 RAD311158
5.2 RAD130198
7.6 RAD101799
7.9 RAD131045
10.9 RAD212359
11.7 RAD302354
13.3 RAD146850
14.6 RAD51523
17.8 RAD187292
19.5 RAD145001
20.1 RAD230684
20.7 RAD160581
22.8 RAD76478
25.7 RAD185006
27.6 RAD264920
28.7 RAD306503
30.7 RAD248822
31.3 RAD189236
32.8 RAD37292
35.0 RAD117897
37.3 RAD202839
37.5 RAD57857
38.3 RAD215516
38.6 RAD25204
38.6 RAD78259
41.3 RAD215529
43.2 RAD134692
44.5 RAD184113
45.3 RAD39175
45.9 RAD218110
46.3 RAD55063
47.7 RAD114986
47.7 RAD80467
49.3 RAD53474
50.2 RAD181114
52.0 RAD128191
52.5 RAD153441
52.9 RAD195044
53.6 RAD68260
54.9 RAD306051
57.1 RAD282277
57.6 RAD141193
57.7 RAD54428
58.2 RAD193218
59.0 RAD113032
60.1 RAD18171
60.3 RAD231575
61.0 RAD10474
61.0 RAD56929
62.0 RAD78457
63.2 RAD135304
64.2 RAD64301
66.6 RAD10942
66.6 RAD289808
67.9 RAD59389
69.9 RAD212383
70.5 RAD106073
74.7 RAD199638
75.4 RAD291851
77.0 RAD55938
79.6 RAD179780
80.3 RAD59099
81.9 RAD28397
83.2 RAD50455
84.1 RAD26842
LG20
0.0 RAD225145
3.1 RAD228878
13.6 RAD94082
15.4 RAD166439
16.5 RAD178327
17.1 RAD234187
18.6 RAD28371
19.7 RAD112653
22.0 RAD322637
26.4 RAD52623
27.3 RAD40250
29.5 RAD126156
30.5 RAD316105
32.4 RAD27440
33.5 RAD307885
34.7 RAD128249
36.9 RAD189213
38.3 RAD259369
38.7 RAD248236
38.8 RAD306755
39.5 RAD31990
40.6 RAD194497
41.4 RAD60028
41.9 RAD20480
42.3 RAD98639
43.6 RAD170799
45.7 RAD218267
45.7 RAD49922
47.6 RAD20409
48.3 RAD102799
48.7 RAD188631
49.0 RAD4171
49.5 RAD41490
50.6 RAD90621
51.1 RAD175787
51.4 RAD312589
52.2 RAD143920
52.2 RAD218960
52.2 RAD321931
52.2 RAD49279
52.4 RAD21343
53.0 RAD105709
53.0 RAD212196
53.0 RAD28661
53.6 RAD188550
55.7 RAD32765
56.2 RAD168634
56.2 RAD61247
57.6 RAD313895
63.5 RAD114700
65.5 RAD216317
66.9 RAD111995
67.6 RAD317107
69.2 RAD216037
73.9 RAD69661
85.3 RAD266379
87.4 RAD89502
91.5 RAD31307

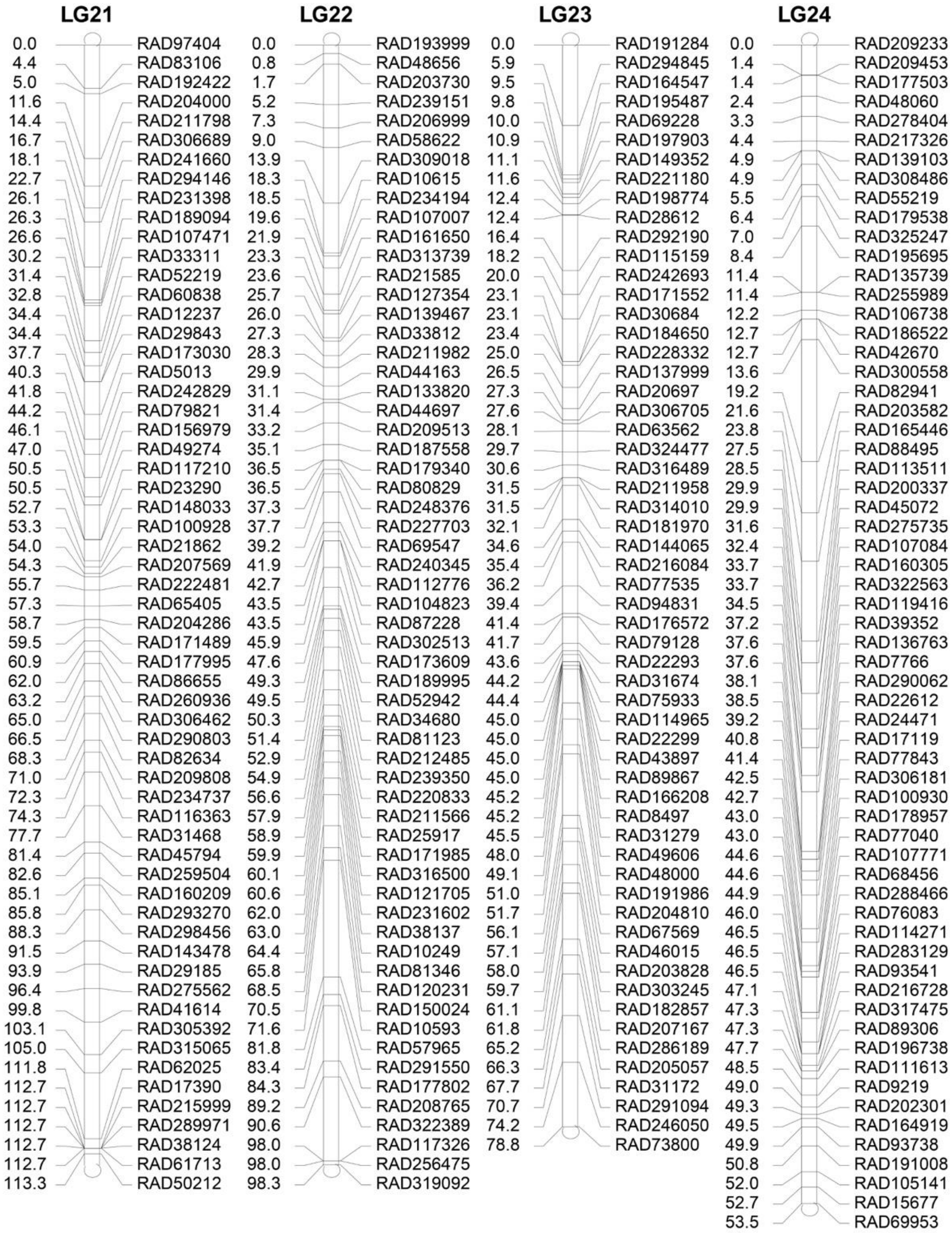

Supplementary Figure S1 The genetic linkage maps of *Megalobrama amblycephala*. Detailed lists of all the molecular markers, including their genetic distance (cM) in each linkage group, are presented.

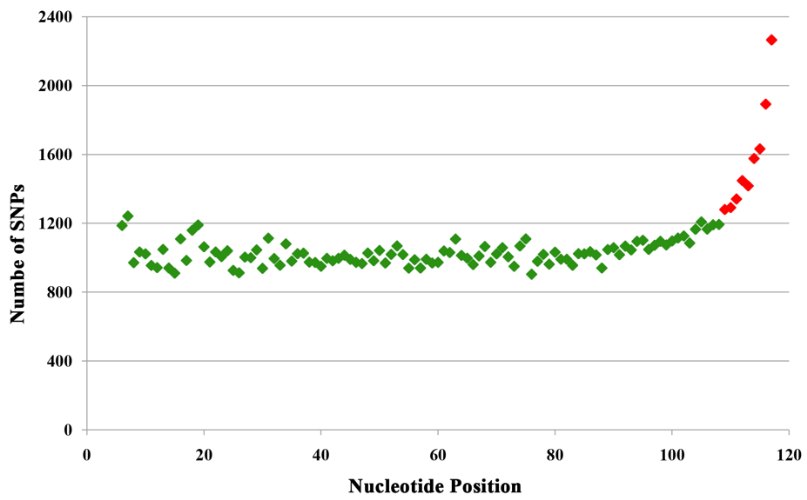

Supplementary Figure S2 Number of SNPs per nucleotide position (1–117). There is an apparent increase in number of SNPs in the last nine nucleotides (109–117), suggestive of sequencing errors, which were consequently removed from the analyses.
